# Serial immune-inflammatory recovery trajectories after acute ischemic stroke: associations with early neurological deterioration and 90-day functional outcome in a retrospective cohort

**DOI:** 10.3389/fneur.2026.1913111

**Published:** 2026-09-17

**Authors:** Ruihan Ni, Lan Chu

**Affiliations:** 1 Suzhou Medical College, Soochow University, Suzhou, Jiangsu, China; 2 Department of Neurology, Affiliated Hospital of Guizhou Medical University, Guiyang, Guizhou, China

**Keywords:** acute ischemic stroke, high-sensitivity C-reactive protein, immune-inflammatory trajectory, latent class mixed model, modified Rankin scale, neutrophil-to-lymphocyte ratio, systemic immune-inflammation index, systemic inflammation

## Abstract

**Background:**

Systemic inflammation after acute ischemic stroke is usually assessed using admission biomarkers, which may not distinguish transient stress from sustained immune-inflammatory activation. We examined whether serial immune-inflammatory recovery trajectories were associated with early neurological deterioration (END) and 90-day functional outcome.

**Methods:**

This single-center retrospective cohort included 760 adults with acute ischemic stroke identified from a hospital stroke database. NLR, SII, SIRI, and hs-CRP measured at admission, 24 h, 72 h, and day 7 were log-transformed and standardized, and equally weighted values were averaged at each observed time point. Latent class mixed models with 2–5 classes were compared without using outcome information, and a five-class solution was selected before association and prediction analyses. The primary outcome was 90-day mRS 3–6. END was defined as an NIHSS increase of ≥2 points within 72 h; the landmark END analysis excluded END within 24 h and tested 0–24 h immune change for END from 24 to 72 h.

**Results:**

The five LCMM classes comprised 182 low-stable, 223 transient moderate, 161 delayed recovery, 122 persistent high, and 72 extreme persistent high patients. Among 751 patients with 90-day outcome data, the observed rate of mRS 3–6 increased from 21.5 to 30.6%, 44.2, 71.9, and 98.6% across these classes. After adjustment, persistent high (OR 2.46, 95% CI 1.31–4.61; *p* = 0.005) and extreme persistent high inflammation (OR 20.24, 95% CI 2.48–165.26; *p* = 0.005) were associated with mRS 3–6; a bias-reduced sensitivity estimate for the extreme class remained large (OR 13.61, 95% CI 2.34–79.26). Ordinal analysis showed progressively worse mRS for delayed recovery (common OR 2.73), persistent high (4.18), and extreme persistent high (6.72). The 0–24 h composite immune-change metric was not associated with landmark END (OR 1.12, 95% CI 0.92–1.36; *p* = 0.265). Adding LCMM-5 to the clinical model increased AUC from 0.828 to 0.841, but did not clearly outperform adding admission NLR/SII.

**Conclusion:**

Longitudinal classification identified five immune-inflammatory recovery trajectories with a marked gradient in 90-day disability. Persistent-high and extreme persistent-high classes retained independent associations with poor functional outcome.

## 1 Introduction

Acute ischemic stroke initiates a rapid dialog between the injured brain and the peripheral immune system. Local innate immune activation, endothelial injury, blood–brain barrier disruption, neutrophil recruitment, platelet activation, lymphocyte suppression, and systemic acute-phase responses may all occur within the first hours to days after vessel occlusion (1, 2). These responses are biologically plausible contributors to infarct progression and secondary complications, but they are also markers of stroke severity and systemic stress (3, 4).

Routine inflammatory markers have therefore attracted sustained interest in stroke prognosis. Higher neutrophil-to-lymphocyte ratio (NLR), systemic immune-inflammation index (SII), systemic inflammation response index (SIRI), and high-sensitivity C-reactive protein (hs-CRP) have been associated with neurological severity, functional disability, mortality, and post-stroke complications in observational cohorts and meta-analyses (5–8). However, most of this literature still treats inflammation as a single admission value. This is a narrow representation of a biological process that is explicitly time-dependent.

Recent studies have also examined composite markers that integrate different inflammatory or nutritional domains, including the monocyte-to-HDL ratio (MHR), C-reactive protein-to-albumin ratio (CAR), C-reactive protein-to-lymphocyte ratio (CLR), and pan-immune-inflammation value (PIV). These markers have shown prognostic associations in ischemic stroke cohorts, but most remain baseline measures rather than longitudinal descriptions of inflammatory recovery.

The metabolic context is also important. Diabetes and acute stress hyperglycemia are common in ischemic stroke and are linked to poor recovery, larger ischemic injury, reperfusion injury, and infection susceptibility (9–12). Hyperglycemia can amplify oxidative stress, endothelial dysfunction, and inflammatory signaling, while acute brain injury can activate hypothalamic–pituitary–adrenal and sympathetic pathways that reshape peripheral immunity (13, 14). From an immune-metabolic perspective, the question is not only whether inflammatory markers are elevated, but whether their recovery pattern reflects a broader immune-metabolic phenotype after stroke.

A longitudinal approach may help separate patients with a short-lived inflammatory response from those with delayed recovery or sustained high immune-inflammatory burden. Group-based trajectory and latent-class frameworks are useful for summarizing repeated biomarker measurements into clinically interpretable phenotypes, although such classifications require careful handling and external validation (15, 16). In acute stroke, dynamic markers may be particularly relevant because early neurological deterioration (END), edema, hemorrhagic transformation, pneumonia, and functional decline often evolve over overlapping but not identical time windows (17, 18).

We therefore investigated serial immune-inflammatory recovery trajectories in a retrospective cohort of patients with acute ischemic stroke. Using routinely available NLR, SII, SIRI, and hs-CRP measurements across admission, 24 h, 72 h, and day 7, we derived longitudinal classes without using outcome information and examined their associations with observed early clinical events and 90-day functional outcome. To avoid exposure-outcome time overlap for END, we also performed a landmark analysis using 0–24 h immune change to evaluate deterioration from 24 to 72 h. Finally, we assessed whether inflammatory dynamics provided incremental apparent prediction value beyond a clinical model.

## 2 Materials and methods

### 2.1 Study design and setting

This was a single-center retrospective longitudinal observational cohort study conducted at the Department of Neurology and Stroke Center, Affiliated Hospital of Guizhou Medical University, China. The study followed the principles of the Declaration of Helsinki and was reported with reference to the STROBE guidance for observational studies (19). The study protocol was approved by the ethics committee of the Affiliated Hospital of Guizhou Medical University (approval number: gzmu2511904). The requirement for written informed consent was waived by the Ethics Committee because this was a retrospective analysis of de-identified routinely collected clinical data.

Consecutive patients with acute ischemic stroke were retrospectively screened from the hospital stroke database covering January 2022 to December 2025. The final analytic dataset comprised 760 patients, of whom 751 had complete 90-day mRS outcome data and were included in the primary functional-outcome analysis.

### 2.2 Participants

Eligible patients were adults aged 18 years or older with acute ischemic stroke confirmed by computed tomography or magnetic resonance imaging, presentation within 24 h after symptom onset, an admission National Institutes of Health Stroke Scale (NIHSS) assessment, admission blood count data, and at least one repeat inflammatory assessment at 24 h or 72 h. Patients were excluded if they had transient ischemic attack, intracerebral hemorrhage, subarachnoid hemorrhage, cerebral venous sinus thrombosis, prestroke mRS greater than 2, pre-admission infection or antibiotic treatment within 7 days, active malignancy, hematologic disease, autoimmune disease, long-term corticosteroid or immunosuppressive therapy, severe hepatic or renal failure, or insufficient exposure or outcome data. Acute stroke management followed contemporary guideline-based care during the study period (20).

Demographic characteristics, vascular risk factors, prestroke mRS, onset-to-door time, stroke severity, TOAST subtype, large vessel occlusion, infarct location, Alberta Stroke Program Early CT Score (ASPECTS), infarct volume, intravenous thrombolysis, endovascular thrombectomy, fasting glucose, lipid profile, estimated glomerular filtration rate, and albumin were extracted from the electronic medical record. NIHSS was used to quantify neurological deficit (21). TOAST subtype and ASPECTS were defined using established criteria (22, 23).

### 2.3 Laboratory measures and immune-inflammatory trajectory classification

Peripheral blood markers were obtained as part of routine clinical care at admission, approximately 24 h, approximately 72 h, and day 7 or before discharge when available. NLR was calculated as neutrophil count divided by lymphocyte count. SII was calculated as neutrophil count multiplied by platelet count and divided by lymphocyte count. SIRI was calculated as neutrophil count multiplied by monocyte count and divided by lymphocyte count. hs-CRP was recorded in mg/L.

NLR, SII, SIRI, and hs-CRP were transformed using log1p and then z-standardized separately for each biomarker using the mean and sample standard deviation pooled across all available patient-time observations from admission through day 7. At each observed sampling point, the four standardized marker values were equally weighted and averaged to form the composite immune-inflammatory burden score. In this dataset the four markers were jointly available or jointly missing at a given time point; therefore, no composite score was calculated from only a subset of the four markers. Equal weighting was used as a transparent, outcome-independent summary rather than estimating marker weights from clinical outcomes.

Longitudinal classes were derived using finite latent class linear mixed models of the composite burden score with categorical time, class-specific mean trajectories, a common Gaussian random intercept, and Gaussian residual error. Patients with partially missing repeated measurements remained in the observed-data maximum-likelihood analysis under a missing-at-random assumption. Models with 2, 3, 4, and 5 classes were each fitted from multiple starting values. Class number was evaluated without access to clinical outcomes using BIC, sample-size-adjusted BIC, entropy, class-specific average posterior probability, minimum class proportion, convergence, and bootstrap stability. Considering these diagnostics together, the five-class solution was selected; it had the lowest BIC (3679.13 vs. 3686.42 for four classes), entropy 0.781, minimum class-specific average posterior probability 0.818, and a smallest class proportion of 9.5%. The five classes were descriptively labeled low-stable recovery, transient moderate response, delayed recovery, persistent high inflammation, and extreme persistent high inflammation after inspection of the estimated trajectories. Model-selection and classification-quality details are reported in Supplementary Tables S1–S3 and Supplementary Figure S1. The four-class solution showed somewhat greater bootstrap assignment stability, whereas the five-class solution had lower BIC in 28 of 30 paired bootstrap resamples; the full model-selection trade-off is reported in Supplementary Table S1. Class number and modal assignments were determined before association and prediction analyses; each patient was assigned to the class with the highest posterior probability. Class assignment did not use fixed biomarker thresholds or deterministic trajectory cutoffs; the fitted class-specific mean profiles are descriptive centroids rather than decision boundaries. Supplementary Table S9 provides the estimated class-specific composite-burden means at admission, 24 h, 72 h, and day 7, together with modal class size and average posterior probability, to facilitate reproducibility. It also reports the pooled log1p means and standard deviations used for biomarker standardization and the fitted random-intercept and residual standard deviations.

### 2.4 Outcomes

The primary outcome was poor functional outcome at 90 days, defined as mRS 3–6. The mRS was also analyzed as an ordinal outcome across the full 0–6 scale, with death coded as 6 (24, 25). Secondary outcomes included END within 72 h, severe END within 72 h, landmark END from 24 to 72 h, pneumonia within 7 days, symptomatic intracranial hemorrhage within 7 days, malignant cerebral edema within 7 days, 90-day death, and length of hospital stay.

END was defined as an increase in NIHSS score of at least 2 points from baseline within 72 h, while severe END used the more stringent threshold of at least 4 points. Because no single END definition is universally accepted, the ≥2-point NIHSS increase was used as the primary threshold because it is a commonly used sensitive definition in acute ischemic stroke cohorts, while ≥4 points was retained as a stricter marker of more substantial neurological deterioration; comparative work has specifically evaluated both thresholds, and recent multicenter studies continue to use ≥2 points for END and ≥4 points for more significant worsening (24–26). For the landmark END analysis, patients who had already developed END within 24 h were specifically excluded, and subsequent END between 24 and 72 h was modeled using only 0–24 h immune-change variables. This time restriction prevented use of 72 h inflammatory measurements to predict an event occurring within the same 72 h window.

### 2.5 Statistical analysis

Continuous variables are summarized as median with interquartile range, and categorical variables as number and percentage. Because many clinical and inflammatory variables were skewed and were summarized using medians and interquartile ranges, between-class comparisons used Kruskal-Wallis tests for continuous variables; Pearson chi-square tests were used for categorical variables. All *p* values were two-sided, and *p* < 0.05 was considered statistically significant. Secondary, interaction, and sensitivity analyses were exploratory; no multiplicity adjustment was applied to these analyses, and their *p* values were interpreted accordingly.

The primary association between LCMM class and 90-day mRS 3–6 was assessed using multivariable logistic regression, with low-stable recovery as the reference. The clinical covariate set comprised age, sex, prestroke mRS, admission NIHSS, large vessel occlusion, log-transformed infarct volume, fasting glucose, intravenous thrombolysis, and endovascular thrombectomy. Fasting glucose was used because it was the consistently defined glycemic variable available in the analytic database; admission glucose was not retained as a uniformly defined source variable for construction of a stress-hyperglycemia ratio. The retrospective database did not retain a uniform clock-time stamp for the fasting sample beyond its designation as fasting glucose, which is acknowledged as a limitation. An extended sensitivity model additionally included landmark END, pneumonia, symptomatic intracranial hemorrhage, and malignant cerebral edema; these post-stroke events were not included in the primary model because they may lie on the pathway between inflammatory recovery and functional outcome.

Ordinal shift analysis of 90-day mRS used ordinal logistic regression to estimate common odds ratios for worse functional status. This approach preserves information across the ordered mRS categories and avoids relying only on a fixed dichotomy (27). For the END landmark models, 0–24 h immune change was calculated as log-transformed ratio change from admission to 24 h and standardized per standard deviation. Separate models were fitted for the composite immune change score, NLR change, SII change, and hs-CRP change, adjusting for the same core clinical covariates where applicable.

Sensitivity analyses for 90-day mRS 3–6 excluded patients with infection within 7 days, excluded patients with symptomatic intracranial hemorrhage or malignant cerebral edema, and evaluated reperfusion-treatment strata. Because the extreme persistent-high class had near-complete separation for the primary binary outcome, a bias-reduced logistic regression sensitivity analysis was used to assess the robustness of the conventional maximum-likelihood estimates. A global LCMM-class × reperfusion interaction term was tested; treatment-stratified estimates were considered exploratory because of nonrandom treatment allocation and sparse cells. A separate 0–72 h k-means analysis was retained as an unsupervised classification sensitivity analysis and compared with the primary LCMM-5 classification in Supplementary material.

Apparent prediction performance was evaluated in a common complete-case population. The clinical model comprised age, sex, prestroke mRS, admission NIHSS, large vessel occlusion, log-transformed infarct volume, fasting glucose, intravenous thrombolysis, and endovascular thrombectomy. This model was compared with extensions adding admission NLR/SII, 0–24 h immune change, or LCMM-5 class. Discrimination was assessed using AUC, prediction error using the Brier score, calibration using predicted-risk deciles, and clinical utility using decision-curve analysis. Paired bootstrap resampling was used to quantify confidence intervals for changes in AUC and Brier score. These analyses represent apparent performance and were not externally validated. LCMM likelihood optimization and bootstrap procedures were implemented in Python 3.13 using NumPy/SciPy-based routines; downstream regression and prediction analyses used statsmodels and scikit-learn, and figures were produced with matplotlib.

## 3 Results

### 3.1 Study population and baseline profile

The full cohort included 760 patients, all of whom contributed to the LCMM trajectory analysis; 751 had complete 90-day functional outcome data. The five classes comprised 182 low-stable recovery (23.9%), 223 transient moderate response (29.3%), 161 delayed recovery (21.2%), 122 persistent high inflammation (16.1%), and 72 extreme persistent high inflammation (9.5%) patients. Overall, the median age was 65.0 years (IQR 58.0–74.0), 481 patients (63.3%) were male, and 333 of 751 patients (44.3%) with available follow-up had 90-day mRS 3–6 (Table 1; Supplementary Tables S2, S3).

**Table 1:** Baseline characteristics according to LCMM-5 class.

Characteristic	Level	Overall	Class 1 Low-stable	Class 2 Transient moderate	Class 3 Delayed recovery	Class 4 Persistent high	Class 5 Extreme persistent high	*p*value
Age, years		65.0 (58.0, 74.0)	62.0 (55.0, 69.0)	64.0 (58.0, 72.0)	65.0 (56.0, 75.0)	68.0 (62.0, 75.8)	72.5 (63.8, 80.2)	<0.001
Body mass index, kg/m2		24.4 (22.0, 26.6)	24.4 (22.4, 26.5)	24.2 (22.0, 26.6)	24.4 (21.8, 26.8)	24.2 (22.1, 26.5)	24.5 (21.8, 26.7)	0.981
Prestroke mRS		0 (0, 0)	0 (0, 0)	0 (0, 0)	0 (0, 0)	0 (0, 0)	0 (0, 0)	0.002
Onset-to-door time, h		4.5 (2.6, 7.4)	4.9 (2.9, 7.6)	4.4 (2.5, 6.8)	4.6 (3.0, 7.5)	4.5 (3.0, 8.1)	3.4 (2.0, 6.6)	0.106
Admission NIHSS		6 (3, 11)	3 (2, 5)	5 (2, 8)	6 (3, 10)	10 (7, 19)	20 (16, 23)	<0.001
ASPECTS		8 (6, 9)	9 (8, 10)	9 (7, 10)	8 (7, 9)	7 (5, 8)	4 (3, 6)	<0.001
Infarct volume, mL		21.0 (9.9, 43.9)	10.6 (6.3, 22.5)	15.9 (8.8, 36.5)	22.6 (11.5, 43.2)	40.0 (20.3, 71.8)	63.9 (37.9, 96.7)	<0.001
Fasting glucose, mmol/L		6.2 (5.4, 7.4)	5.9 (5.1, 6.9)	6.2 (5.2, 7.3)	6.4 (5.5, 7.6)	6.6 (5.8, 8.0)	6.8 (5.6, 8.1)	<0.001
LDL-C, mmol/L		2.7 (2.2, 3.2)	2.5 (2.2, 3.2)	2.8 (2.3, 3.3)	2.7 (2.3, 3.2)	2.8 (2.3, 3.3)	2.7 (2.2, 3.2)	0.187
eGFR, mL/min/1.73 m2		83.5 (74.2, 92.2)	85.7 (77.2, 94.8)	84.7 (75.5, 91.6)	83.3 (75.5, 93.0)	81.1 (72.6, 89.9)	79.1 (68.7, 85.0)	<0.001
Admission albumin, g/L		39.0 (36.8, 41.0)	39.6 (37.8, 41.3)	39.4 (37.0, 41.5)	38.9 (36.8, 40.9)	38.0 (35.3, 40.4)	37.7 (35.3, 39.9)	<0.001
Male sex		481 (63.3%)	113 (62.1%)	140 (62.8%)	103 (64.0%)	78 (63.9%)	47 (65.3%)	0.989
Rural residence		347 (45.7%)	74 (40.7%)	108 (48.4%)	78 (48.4%)	57 (46.7%)	30 (41.7%)	0.471
Hypertension		286 (37.6%)	62 (34.1%)	83 (37.2%)	61 (37.9%)	50 (41.0%)	30 (41.7%)	0.719
Diabetes mellitus		141 (18.6%)	28 (15.4%)	30 (13.5%)	32 (19.9%)	35 (28.7%)	16 (22.2%)	0.007
Dyslipidemia		295 (38.8%)	73 (40.1%)	91 (40.8%)	61 (37.9%)	47 (38.5%)	23 (31.9%)	0.737
Atrial fibrillation		53 (7.0%)	11 (6.0%)	12 (5.4%)	8 (5.0%)	11 (9.0%)	11 (15.3%)	0.032
Prior stroke/TIA		122 (16.1%)	28 (15.4%)	34 (15.2%)	23 (14.3%)	20 (16.4%)	17 (23.6%)	0.462
Current smoking		290 (38.2%)	63 (34.6%)	87 (39.0%)	72 (44.7%)	42 (34.4%)	26 (36.1%)	0.306
Large vessel occlusion		266 (35.0%)	14 (7.7%)	48 (21.5%)	69 (42.9%)	67 (54.9%)	68 (94.4%)	<0.001
Anterior circulation infarct		589 (77.5%)	139 (76.4%)	173 (77.6%)	119 (73.9%)	99 (81.1%)	59 (81.9%)	0.547
Intravenous thrombolysis		263 (34.6%)	59 (32.4%)	77 (34.5%)	60 (37.3%)	42 (34.4%)	25 (34.7%)	0.926
Endovascular thrombectomy		258 (33.9%)	30 (16.5%)	63 (28.3%)	55 (34.2%)	52 (42.6%)	58 (80.6%)	<0.001
TOAST subtype	Cardioembolism	191 (25.1%)	29 (15.9%)	53 (23.8%)	33 (20.5%)	40 (32.8%)	36 (50.0%)	<0.001
TOAST subtype	Large-artery atherosclerosis	308 (40.5%)	69 (37.9%)	77 (34.5%)	75 (46.6%)	58 (47.5%)	29 (40.3%)	
TOAST subtype	Other determined	26 (3.4%)	6 (3.3%)	6 (2.7%)	7 (4.3%)	3 (2.5%)	4 (5.6%)	
TOAST subtype	Small-vessel occlusion	193 (25.4%)	70 (38.5%)	69 (30.9%)	34 (21.1%)	18 (14.8%)	2 (2.8%)	
TOAST subtype	Undetermined	42 (5.5%)	8 (4.4%)	18 (8.1%)	12 (7.5%)	3 (2.5%)	1 (1.4%)	

Values are median (IQR) or *n* (%), based on the full cohort (*N* = 760). Because many continuous variables were skewed, p values were calculated using Kruskal-Wallis tests for continuous variables and Pearson chi-square tests for categorical variables. mRS, modified Rankin Scale; NIHSS, National Institutes of Health Stroke Scale; LVO, large vessel occlusion; ASPECTS, Alberta Stroke Program Early CT Score; LDL-C, low-density lipoprotein cholesterol; eGFR, estimated glomerular filtration rate; TIA, transient ischemic attack.

Clinical severity increased across the higher-burden classes. Median admission NIHSS increased from 3 in the low-stable class to 20 in the extreme persistent-high class; large vessel occlusion increased from 7.7 to 94.4%, and median infarct volume from 10.6 to 63.9 mL. Endovascular thrombectomy was performed in 16.5% of the low-stable class and 80.6% of the extreme persistent-high class. Fasting glucose also increased across classes from a median of 5.9 to 6.8 mmol/L (Table 1).

### 3.2 Serial inflammatory recovery patterns and observed clinical events

The five LCMM trajectories showed clear longitudinal separation (Figure 1A). Low-stable recovery remained at low composite burden throughout follow-up; transient moderate response showed an early increase followed by decline; delayed recovery increased through approximately 72 h before partial recovery by day 7; persistent high inflammation remained elevated; and extreme persistent high inflammation remained markedly elevated across the serial measurements. Individual NLR, SII, SIRI, and hs-CR*p* values showed corresponding separation (Supplementary Table S10). Within each time point, NLR, SII, and SIRI were strongly correlated (Spearman ρ 0.883–0.979), whereas correlations with hs-CRP were lower (ρ 0.682–0.864), indicating substantial overlap among the blood-cell-derived indices and comparatively distinct information from hs-CRP (Supplementary Table S4; Supplementary Figure S2).

**Figure 1:**
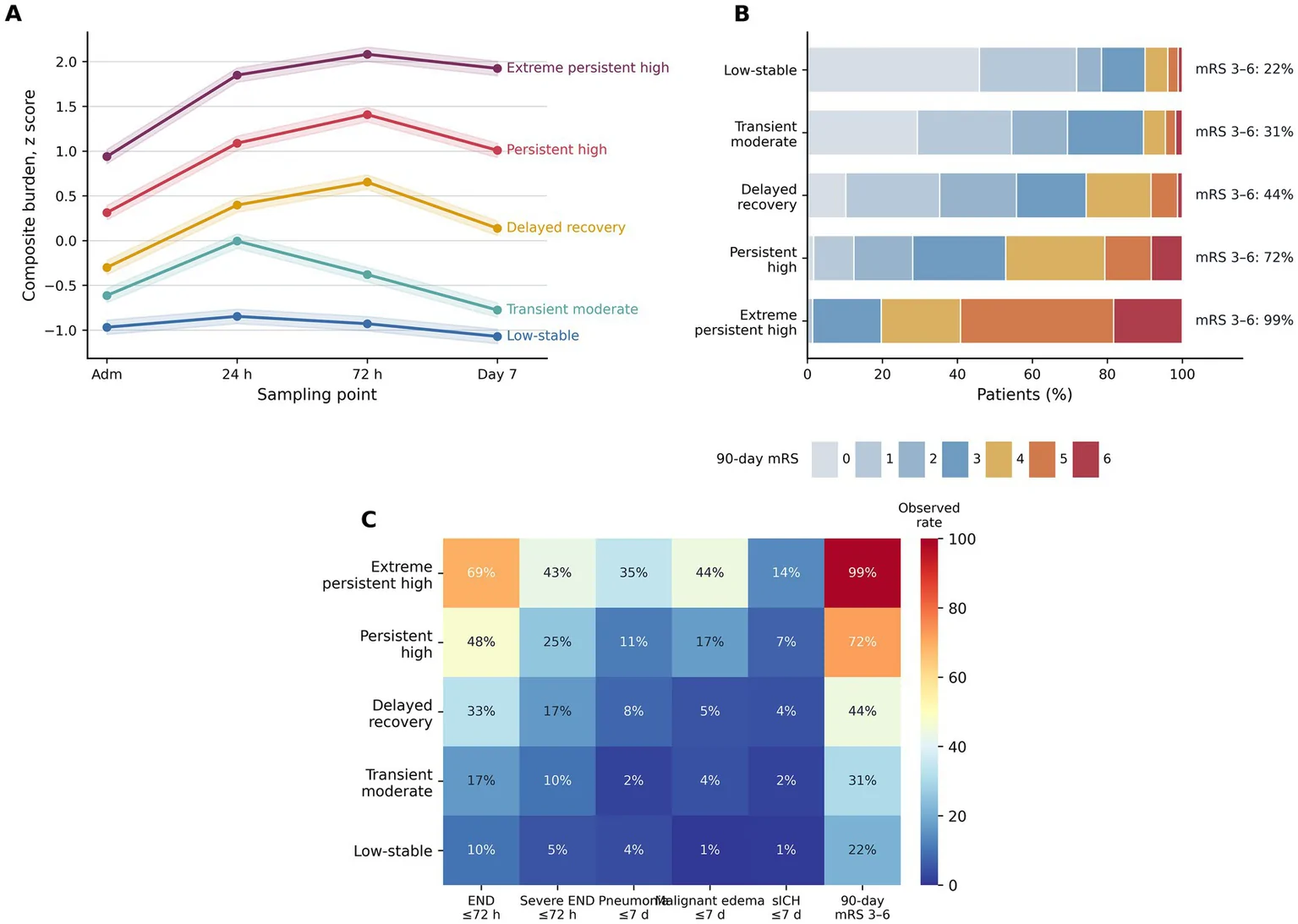
LCMM-5 trajectory structure and observed event patterns. **(A)** Shows the mean composite immune-inflammatory burden across admission, 24 h, 72 h, and day 7 for the five LCMM classes. **(B)** Shows the distribution of 90-day modified Rankin Scale (mRS) scores across LCMM-5 classes, together with the proportion of patients with mRS 3–6. **(C)** Shows observed rates of END, severe END, pneumonia, malignant edema, sICH, and 90-day mRS 3–6 across classes. Event rates are descriptive and unadjusted. END, early neurological deterioration; hs-CRP, high-sensitivity C-reactive protein; mRS, modified Rankin Scale; NLR, neutrophil-to-lymphocyte ratio; SII, systemic immune-inflammation index; SIRI, systemic inflammation response index; sICH, symptomatic intracranial hemorrhage.

Observed clinical events followed a strong gradient across classes (Figures 1B,C; Table 2). END within 72 h increased from 9.9% in low-stable recovery to 69.4% in extreme persistent high inflammation; severe END increased from 4.9 to 43.1%, pneumonia from 3.8 to 34.7%, malignant cerebral edema from 0.5 to 44.4%, and symptomatic intracranial hemorrhage from 1.1 to 13.9%. The observed proportion with 90-day mRS 3–6 was 21.5, 30.6, 44.2, 71.9, and 98.6% across classes 1–5, respectively. Ninety-day mortality increased from 1.1 to 18.3%. These event rates are descriptive and unadjusted.

**Table 2:** Observed clinical outcomes by LCMM-5 class.

Characteristic	Overall	Class 1 Low-stable	Class 2 Transient moderate	Class 3 Delayed recovery	Class 4 Persistent high	Class 5 Extreme persistent high	*p* value
90-day mRS	2 (1, 4)	1 (0, 2)	1 (0, 3)	2 (1, 4)	3 (2, 4)	5 (4, 5)	<0.001
Length of stay, d	11.0 (8.0, 14.0)	10.0 (8.0, 12.0)	11.0 (8.0, 13.0)	11.0 (9.0, 14.0)	13.0 (10.0, 16.0)	16.0 (13.0, 19.0)	<0.001
END within 72 h	216 (28.4%)	18 (9.9%)	37 (16.6%)	53 (32.9%)	58 (47.5%)	50 (69.4%)	<0.001
Severe END within 72 h	120 (15.8%)	9 (4.9%)	22 (9.9%)	27 (16.8%)	31 (25.4%)	31 (43.1%)	<0.001
Pneumonia within 7 d	64 (8.4%)	7 (3.8%)	5 (2.2%)	13 (8.1%)	14 (11.5%)	25 (34.7%)	<0.001
Symptomatic ICH within 7 d	33 (4.3%)	2 (1.1%)	5 (2.2%)	7 (4.3%)	9 (7.4%)	10 (13.9%)	<0.001
Malignant cerebral edema within 7 d	72 (9.5%)	1 (0.5%)	10 (4.5%)	8 (5.0%)	21 (17.2%)	32 (44.4%)	<0.001
90-day mRS 3–6	333 (44.3%)	39 (21.5%)	68 (30.6%)	69 (44.2%)	87 (71.9%)	70 (98.6%)	<0.001
90-day death	31 (4.1%)	2 (1.1%)	4 (1.8%)	2 (1.3%)	10 (8.3%)	13 (18.3%)	<0.001

Values are median (IQR) or *n* (%). Event rates are unadjusted descriptive proportions. The main table is restricted to clinically interpretable outcomes; full serial NLR, SII, SIRI, and hs-CRP distributions are reported in Supplementary Table S10. Full ordinal mRS distributions are shown in Figure 1B; mRS 3–6 was the prespecified primary functional outcome. END, early neurological deterioration; mRS, modified Rankin Scale; sICH, symptomatic intracranial hemorrhage.

### 3.3 Adjusted associations with 90-day functional outcome

In the primary adjusted logistic model, LCMM class was associated with 90-day mRS 3–6 overall (global *p* < 0.001). Compared with low-stable recovery, transient moderate response (OR 0.94, 95% CI 0.57–1.56; *p* = 0.810) and delayed recovery (OR 1.35, 95% CI 0.78–2.32; *p* = 0.278) were not independently associated with the dichotomized outcome, whereas persistent high inflammation (OR 2.46, 95% CI 1.31–4.61; *p* = 0.005) and extreme persistent high inflammation (OR 20.24, 95% CI 2.48–165.26; *p* = 0.005) were associated with higher odds of mRS 3–6 (Figure 2A; Table 3). Because only one patient in the extreme persistent-high class had mRS 0–2, a bias-reduced sensitivity model was also fitted and yielded a directionally consistent estimate (OR 13.61, 95% CI 2.34–79.26; Supplementary Table S5).

**Figure 2:**
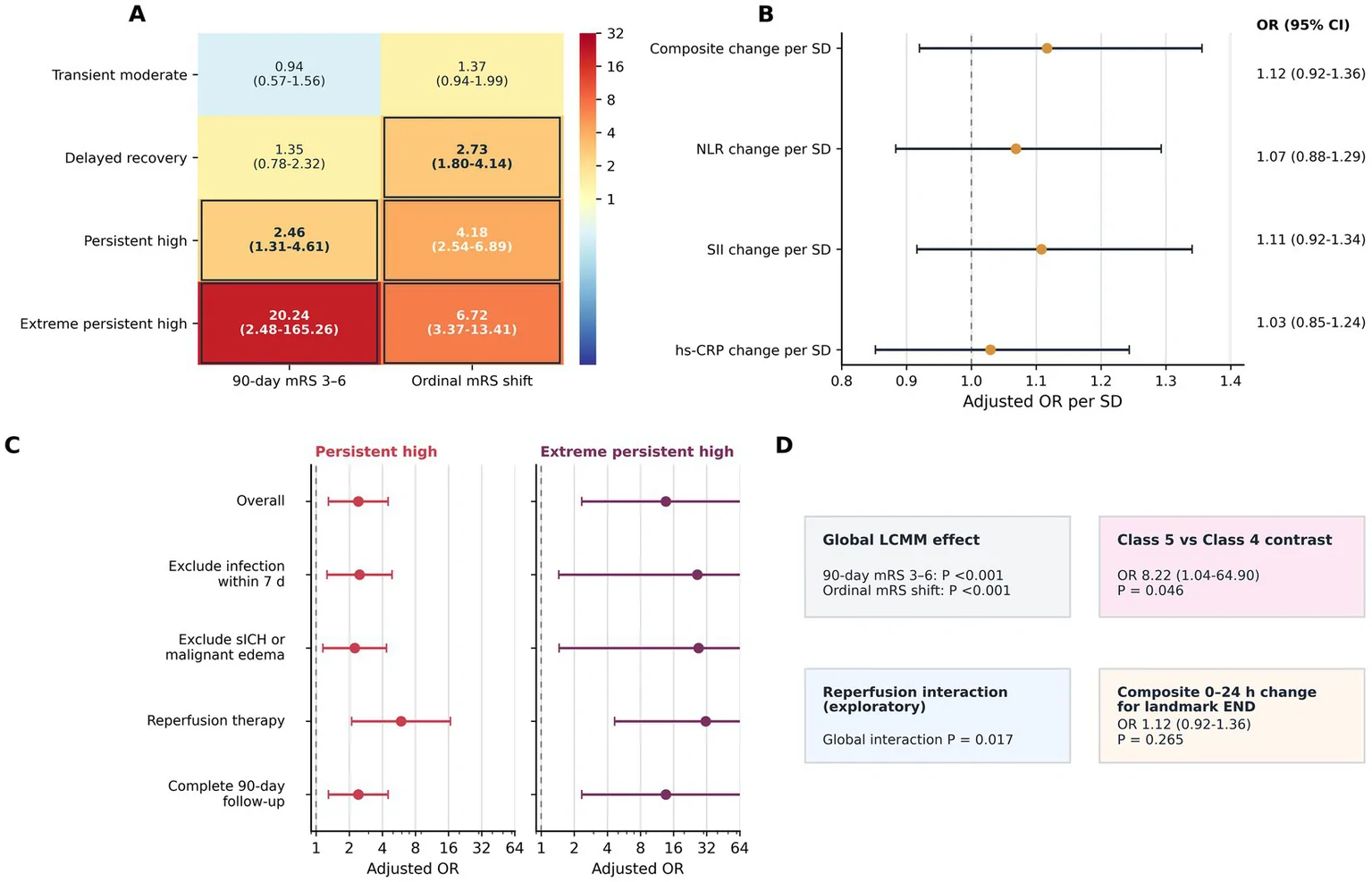
Adjusted associations and sensitivity analyses. **(A)** Summarizes adjusted effect sizes for the non-reference LCMM-5 classes for 90-day mRS 3–6 and ordinal mRS shift. **(B)** Shows the landmark analysis for END from 24 to 72 h according to 0–24 h immune-change metrics after excluding END within 24 h. **(C)** Shows restricted-sample sensitivity analyses for persistent-high and extreme persistent-high classes using bias-reduced logistic regression; exact numerical estimates are provided in Supplementary Table S5. **(D)** Summarizes the global LCMM effect, the class 5 versus class 4 contrast, the exploratory reperfusion interaction, and the composite 0–24 h change association for landmark END. END, early neurological deterioration; hs-CRP, high-sensitivity C-reactive protein; mRS, modified Rankin Scale; NLR, neutrophil-to-lymphocyte ratio; OR, odds ratio; SII, systemic immune-inflammation index; sICH, symptomatic intracranial hemorrhage.

**Table 3:** Adjusted associations with 90-day outcomes and landmark END.

Analysis	Comparison	Effect measure	Adjusted OR (95% CI)	*p* value
90-day mRS 3–6	Transient moderate response vs. Low-stable recovery	Adjusted OR	0.94 (0.57–1.56)	0.810
90-day mRS 3–6	Delayed recovery vs. Low-stable recovery	Adjusted OR	1.35 (0.78–2.32)	0.278
90-day mRS 3–6	Persistent high inflammation vs. Low-stable recovery	Adjusted OR	2.46 (1.31–4.61)	0.005
90-day mRS 3–6	Extreme persistent high inflammation vs. Low-stable recovery	Adjusted OR	20.24 (2.48–165.26)	0.005
Ordinal 90-day mRS shift	Transient moderate response vs. Low-stable recovery	Common OR	1.37 (0.94–1.99)	0.104
Ordinal 90-day mRS shift	Delayed recovery vs. Low-stable recovery	Common OR	2.73 (1.80–4.14)	<0.001
Ordinal 90-day mRS shift	Persistent high inflammation vs. Low-stable recovery	Common OR	4.18 (2.54–6.89)	<0.001
Ordinal 90-day mRS shift	Extreme persistent high inflammation vs. Low-stable recovery	Common OR	6.72 (3.37–13.41)	<0.001
Landmark END 24–72 h	Composite immune change per SD	Adjusted OR per SD	1.12 (0.92–1.36)	0.265
Landmark END 24–72 h	NLR change per SD	Adjusted OR per SD	1.07 (0.88–1.29)	0.494
Landmark END 24–72 h	SII change per SD	Adjusted OR per SD	1.11 (0.92–1.34)	0.291
Landmark END 24–72 h	hs-CRP change per SD	Adjusted OR per SD	1.03 (0.85–1.24)	0.765

Low-stable recovery was the reference LCMM class. Primary and ordinal outcome models were adjusted for age, sex, prestroke mRS, admission NIHSS, large vessel occlusion, log infarct volume, fasting glucose, intravenous thrombolysis, and endovascular thrombectomy. The landmark END models excluded patients with END within 24 h. Bias-reduced sensitivity estimates for sparse class 5 cells are provided in Supplementary Table S5.

In ordinal shift analysis, the association showed a clearer graded pattern. Compared with low-stable recovery, the common ORs for a worse mRS category were 1.37 (95% CI 0.94–1.99; *p* = 0.104) for transient moderate response, 2.73 (95% CI 1.80–4.14; *p* < 0.001) for delayed recovery, 4.18 (95% CI 2.54–6.89; *p* < 0.001) for persistent high inflammation, and 6.72 (95% CI 3.37–13.41; *p* < 0.001) for extreme persistent high inflammation (global *p* < 0.001; Figure 2A; Table 3).

In the extended sensitivity model including landmark END, pneumonia, symptomatic intracranial hemorrhage, and malignant cerebral edema, the estimates were attenuated for classes 2–4, whereas the extreme persistent-high class remained associated with 90-day mRS 3–6 (OR 13.12, 95% CI 1.57–109.87; *p* = 0.018). This model is interpreted cautiously because these early complications may lie on the pathway between inflammatory recovery and functional outcome (Table 3).

### 3.4 Landmark END analysis and sensitivity analyses

The landmark END analysis included 625 complete cases after excluding patients who developed END within 24 h. In this time-restricted analysis, 0–24 h composite immune change was not independently associated with END from 24 to 72 h (OR per SD 1.12, 95% CI 0.92–1.36; *p* = 0.265). Similar null findings were observed for NLR change (OR 1.07, 95% CI 0.88–1.29; *p* = 0.494), SII change (OR 1.11, 95% CI 0.92–1.34; *p* = 0.291), and hs-CRP change (OR 1.03, 95% CI 0.85–1.24; *p* = 0.765; Figure 2B; Table 3).

Bias-reduced sensitivity analyses supported the robustness of the higher-risk classes across clinically relevant restrictions (Figure 2C; Supplementary Table S5). The global LCMM-class × reperfusion interaction test was statistically significant (Figure 2D; P for interaction = 0.017), but the treatment-stratified results are considered exploratory because reperfusion treatment was nonrandom and the extreme persistent-high class was sparse in some strata. The no-reperfusion estimates are therefore presented only in the Supplementary material.

The 0–72 h k-means sensitivity classification showed a strong ordered relationship with the LCMM-5 solution (Spearman = 0.932), with most disagreement occurring between adjacent higher-burden classes. This analysis was used only as a classification robustness check and did not determine the primary class number or patient assignments (Supplementary Table S6; Supplementary Figure S3).

### 3.5 Apparent prediction performance

Prediction analyses were performed in a common complete-case population of 692 patients with 311 outcome events. The clinical model achieved an AUC of 0.828 (95% bootstrap CI 0.797–0.858) and a Brier score of 0.165. Adding admission NLR/SII yielded an AUC of 0.840 and Brier score of 0.159; adding 0–24 h immune change yielded an AUC of 0.829 and Brier score of 0.165; and adding LCMM-5 yielded an AUC of 0.841 and Brier score of 0.158 (Figures 3A,B; Table 4). Relative to the clinical model, LCMM-5 increased AUC by 0.013 (95% paired-bootstrap CI 0.003–0.023) and yielded a ΔBrier of −0.007 (95% CI − 0.012 to −0.002).

**Figure 3:**
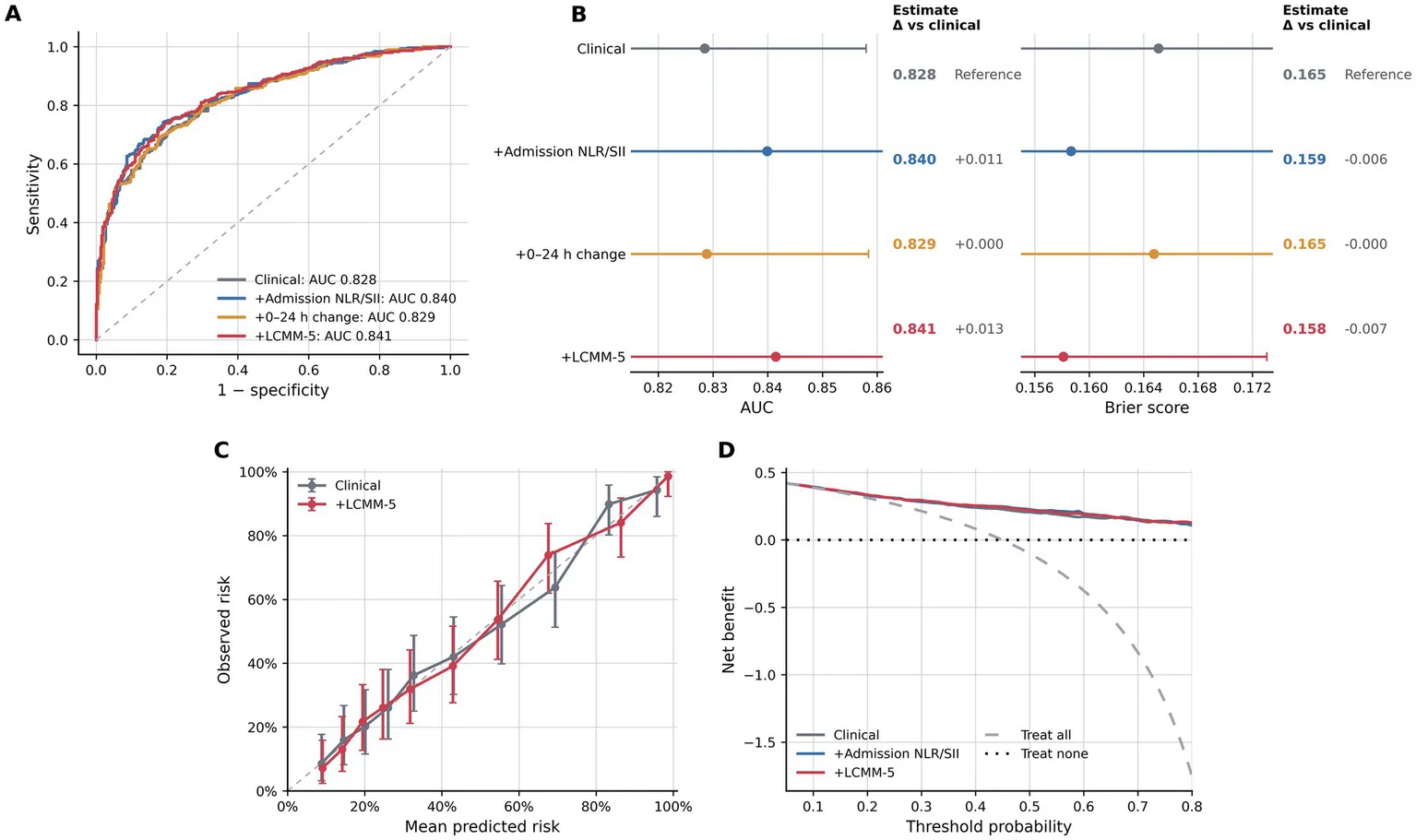
Apparent prediction performance for 90-day poor functional outcome. **(A)** Shows ROC curves for the clinical model and models additionally incorporating admission NLR/SII, 0–24 h immune change, or LCMM-5. **(B)** Summarizes AUC and Brier score with bootstrap confidence intervals and changes relative to the clinical model. **(C)** Shows apparent calibration by predicted-risk deciles for the clinical model and the model including LCMM-5. **(D)** Shows decision-curve analysis across threshold probabilities. All prediction analyses used the same complete-case population and were not externally validated. AUC, area under the receiver operating characteristic curve; mRS, modified Rankin Scale; NLR, neutrophil-to-lymphocyte ratio; SII, systemic immune-inflammation index. The clinical model comprised age, sex, prestroke mRS, admission NIHSS, large vessel occlusion, log-transformed infarct volume, fasting glucose, intravenous thrombolysis, and endovascular thrombectomy.

**Table 4:** Apparent prediction performance for 90-day mRS 3–6.

Model	AUC (95% bootstrap CI)	ΔAUC vs clinical	Brier (95% bootstrap CI)	ΔBrier vs clinical	AIC
Clinical model	0.828 (0.797–0.858)	Reference	0.165 (0.150–0.180)	Reference	711.2
Clinical + admission NLR/SII	0.840 (0.810–0.869)	+0.011 (+0.000 to +0.023)	0.159 (0.144–0.174)	−0.006 (−0.012 to −0.001)	692.2
Clinical + 0–24 h immune change	0.829 (0.798–0.858)	+0.000 (−0.004 to +0.005)	0.165 (0.150–0.180)	−0.000 (−0.002 to +0.001)	711.6
Clinical + LCMM-5	0.841 (0.811–0.870)	+0.013 (+0.003 to +0.023)	0.158 (0.143–0.173)	−0.007 (−0.012 to −0.002)	694.1

All prediction models were evaluated in the same complete-case population. The clinical model included age, sex, prestroke mRS, admission NIHSS, large vessel occlusion, log infarct volume, fasting glucose, intravenous thrombolysis, and endovascular thrombectomy. Delta AUC and delta Brier were calculated relative to the clinical model with paired-bootstrap confidence intervals. Lower Brier scores indicate lower prediction error. These are apparent performance estimates without external validation.

Calibration by risk decile was visually similar between the clinical and the model including LCMM-5 (Figure 3C), and decision-curve analysis showed broadly overlapping net-benefit curves across most threshold probabilities (Figure 3D). Direct comparison with the model adding admission NLR/SII showed little additional improvement from LCMM-5 (AUC 0.002, 95% CI − 0.010 to 0.012; Brier −0.001, 95% CI − 0.006 to 0.005) (Supplementary Table S8). Thus, serial trajectory classification provided modest apparent improvement over the clinical model but did not clearly outperform simpler admission inflammatory markers.

## 4 Discussion

### 4.1 Principal findings

In this retrospective cohort of patients with acute ischemic stroke, longitudinal modeling identified five immune-inflammatory recovery trajectories with a marked gradient in stroke severity and observed outcomes. Persistent high and extreme persistent high inflammation were independently associated with 90-day mRS 3–6, while delayed recovery showed a more consistent association with worse outcome across the full ordinal mRS distribution than with the dichotomized primary endpoint. The extreme persistent-high class was also characterized by very high baseline stroke severity, large-vessel occlusion, and infarct volume, so the magnitude of its adjusted association should not be interpreted as a causal effect of inflammation.

A second finding is equally important. When the analysis was restricted to a landmark design that excluded END within 24 h, 0–24 h immune change did not independently explain END from 24 to 72 h. This result prevents overinterpretation of early inflammatory change as a direct predictor of subsequent neurological worsening and illustrates the importance of matching biomarker windows to outcome timing.

### 4.2 Relationship to previous inflammatory biomarker studies

Our results extend, rather than simply repeat, the established literature on admission inflammatory markers. Previous studies have linked high NLR with mortality and poor functional outcome after stroke (5), SII with 3-month outcome after intravenous thrombolysis (6), SIRI with mortality and functional prognosis (8, 28), and hs-CRP with death, recurrence, and poor prognosis (7). These studies support the general prognostic relevance of systemic inflammation, but they mostly use a single time point. The substantial correlations among NLR, SII, and SIRI also indicate that these indices are partly redundant rather than independent inflammatory signals; the lower correlations with hs-CRP suggest somewhat more distinct acute-phase information.

The trajectory approach adds a clinically intuitive distinction between transient activation, delayed recovery, and persistent inflammatory burden. In the five-class solution, the upper persistent-inflammatory stratum separated into persistent-high and extreme persistent-high classes. The delayed-recovery class was associated with worse ordinal mRS but not independently with dichotomized mRS 3–6, whereas the two persistent-high classes carried the clearest adjusted associations. This pattern argues for interpreting the trajectories as graded prognostic phenotypes rather than deterministic categories. The fifth class should therefore be interpreted primarily as an intensity refinement within the persistent-inflammatory pattern rather than as a wholly distinct temporal shape.

Dynamic inflammatory analyses are beginning to appear in reperfusion-treated stroke populations. A meta-analysis focused on dynamic NLR after reperfusion therapy suggested that temporal changes in NLR can carry prognostic information (29), and a recent study of SII after thrombolysis reported that early increase in SII was associated with unfavorable 3-month outcome (30). Our findings are compatible with this broader direction but also highlight a methodological boundary: dynamic metrics should be matched to outcome timing. The negative landmark END result in our study is a useful reminder that time-overlap bias can generate misleading claims if 72 h biomarker values are used to predict deterioration occurring within the same 72 h window.

### 4.3 Possible biological interpretation

Persistent high inflammation after stroke may represent an integrated phenotype of severe neurovascular injury, systemic stress, and impaired inflammatory resolution. Neutrophils can contribute to microvascular obstruction, endothelial damage, reactive oxygen species production, and blood–brain barrier disruption, whereas lymphopenia may reflect stroke-induced neuroendocrine and autonomic immune suppression (3, 4). Platelet–neutrophil interactions and acute-phase responses can further amplify thromboinflammation in the ischemic microcirculation (2).

The observed association with pneumonia and malignant edema is biologically plausible but should not be read as proof of mediation. Severe stroke can simultaneously drive a stronger inflammatory response, higher aspiration risk, impaired consciousness, larger infarct volume, edema, and worse functional recovery. Stroke-induced immunodepression, characterized partly by lymphocyte suppression and neuroendocrine activation, has been proposed as one mechanism linking severe brain injury to infection susceptibility (13, 14). Preventive antibiotic strategies have not consistently improved functional outcome, which reinforces that infection is only one component of a more complex post-stroke immune state (31).

The metabolic findings fit this interpretation. Patients in the persistent-high and extreme persistent-high classes had higher metabolic and clinical severity profiles, including higher fasting glucose, and fasting glucose was retained as a covariate in the full model. Acute hyperglycemia can worsen ischemic injury through oxidative stress, endothelial dysfunction, blood–brain barrier damage, and reduced reperfusion efficiency (9, 10). Recent meta-analytic evidence further supports stress hyperglycemia ratio as a marker of poor prognosis in acute ischemic stroke (12). In this context, persistent inflammation may be a vascular-neuroimmune-metabolic phenotype rather than a purely hematologic signal.

### 4.4 Clinical and methodological implications

The clinical attraction of this approach is that it uses tests that are commonly available during acute stroke hospitalization. However, serial classification requires repeated blood sampling, reliable timing, data processing, and delayed availability of a complete trajectory. The apparent prediction gain over a clinical model was modest and was not clearly greater than that achieved by adding admission NLR/SII alone. Accordingly, the present data do not establish that the incremental information from repeated testing justifies additional cost or logistical burden in routine practice. The more defensible role is hypothesis-generating risk stratification and monitoring, pending prospective workflow studies and external validation.

The prediction analysis should also be interpreted conservatively. Adding admission NLR/SII or LCMM-5 to the clinical model produced only small apparent improvements in AUC and Brier score, and LCMM-5 did not clearly outperform the simpler admission NLR/SII extension. These increments are not negligible, but they are not sufficient to claim clinical deployment. TRIPOD and PROBAST guidance emphasize that prediction models require transparent reporting, appropriate validation, and assessment of bias before clinical use (32, 33). Calibration and decision-curve behavior are at least as important as discrimination, especially when model outputs might guide treatment thresholds (34, 35).

The present results therefore support inflammatory recovery trajectories as prognostic phenotypes, not as finalized prediction tools or treatment targets. Future multicenter work should determine whether the five-class structure is reproducible across ethnic groups, healthcare systems, laboratory platforms, reperfusion practices, and different onset-to-door distributions, and whether serial profiling provides clinically meaningful net benefit after internal optimism correction and external validation.

### 4.5 Strengths and limitations

The study has several strengths. It used serial inflammatory measurements rather than only admission values, derived classes without using outcome information, retained patients with partially missing repeated biomarker measurements in the LCMM observed-data likelihood, preserved the full ordinal mRS distribution in a secondary analysis, and used a landmark END design to avoid a major time-ordering problem. The outcome models adjusted for key clinical severity variables including admission NIHSS, large vessel occlusion, infarct volume, fasting glucose, and reperfusion therapies, and bias-reduced sensitivity analyses were used where sparse outcome cells produced near-separation.

Several limitations need to be acknowledged. First, this was a single-center retrospective observational study, so residual confounding, selection bias, center-specific practice patterns, and retrospective event ascertainment cannot be excluded. In particular, the inflammatory classes were strongly correlated with admission NIHSS, large vessel occlusion, infarct volume, and treatment intensity; adjustment cannot fully separate inflammation from the severity and systemic-stress response to the index stroke. Second, inflammatory indices are nonspecific and may reflect stroke severity, metabolic stress, occult infection, treatment effects, or comorbidity rather than a distinct inflammatory pathway. Third, repeated inflammatory measurements were incomplete, especially at day 7; the LCMM retained partially observed trajectories under a missing-at-random assumption, which remains unverifiable. Fourth, the retrospective database recorded fasting glucose as a fasting value but did not retain a uniform sampling timestamp, and admission glucose was not uniformly available for a stress-hyperglycemia ratio analysis. Fifth, the median onset-to-door time was 4.5 h, which may differ from faster reperfusion pathways and can influence the biological timing of inflammatory measurements. Sixth, the cohort was drawn from a single Chinese center, and ethnic background, healthcare access, reperfusion patterns, laboratory practice, and supportive-care pathways may limit transportability to other populations. Finally, prediction performance was apparent and requires internal optimism correction and external validation before clinical use.

## 5 Conclusion

Serial immune-inflammatory recovery trajectories provide a clinically interpretable description of systemic response after acute ischemic stroke. Persistent-high and extreme persistent-high classes were independently associated with 90-day disability, while delayed recovery showed a clearer graded association in ordinal mRS analysis. Early 0–24 h immune change did not independently predict landmark END from 24 to 72 h, and the incremental prediction gain of LCMM-5 beyond admission inflammatory markers was small. Multicenter external validation is required before clinical implementation.

## Data Availability

The original contributions presented in the study are included in the article/Supplementary material, further inquiries can be directed to the corresponding author/s.
