# Supplementary material for: Serial immune-inflammatory recovery trajectories after acute ischemic stroke: associations with early neurological deterioration and 90-day functional outcome in a retrospective cohort
Source: Front Neurol. 2026 Sep 17;17:1913111. doi: 10.3389/fneur.2026.1913111 (PMC13627095; doi:10.3389/fneur.2026.1913111)
Supplement: Supplementary file 1 [file Supplementary_file_1.docx]

**Supplementary Materials**

**Serial immune-inflammatory recovery trajectories after acute ischemic stroke: associations with early neurological deterioration and 90-day functional outcome in a retrospective cohort**

# Supplementary Tables

## Supplementary Table S1. Latent class mixed-model selection

| **Classes** | **Log-likelihood** | **AIC** | **BIC** | **SABIC** | **Entropy** | **Mean maximum posterior probability** | **Minimum class-specific APP** | **Smallest modal class, %** |
| --- | --- | --- | --- | --- | --- | --- | --- | --- |
| 2 | -1882.90 | 3787.80 | 3838.76 | 3803.83 | 0.850 | 0.958 | 0.940 | 40.0 |
| 3 | -1820.61 | 3673.23 | 3747.36 | 3696.56 | 0.833 | 0.925 | 0.863 | 16.7 |
| 4 | -1773.56 | 3589.12 | 3686.42 | 3619.74 | 0.755 | 0.856 | 0.812 | 17.1 |
| 5 | -1753.33 | 3558.67 | 3679.13 | 3596.57 | 0.781 | 0.851 | 0.818 | 9.5 |

Models with 2–5 classes were fitted without using clinical outcomes. The five-class model was selected based on overall fit, classification quality, minimum class size, convergence, and stability. Among the two leading solutions, 50-resample bootstrap mean adjusted Rand index/exact assignment agreement were 0.883/95.4% for the four-class model and 0.826/90.3% for the five-class model; in 30 paired bootstrap resamples, the five-class model had the lower BIC in 28 (93.3%). Thus, although the four-class solution showed somewhat greater assignment stability, the five-class solution was favored by the overall model-selection evidence. APP, average posterior probability; BIC, Bayesian information criterion; SABIC, sample-size-adjusted BIC. A sensitivity specification allowing time-specific residual variances also retained the five-class solution as BIC-preferred.

## Supplementary Table S2. Posterior classification quality for the selected five-class LCMM solution

| **LCMM class** | **Modal n** | **Modal %** | **Estimated class probability** | **Average posterior probability** |
| --- | --- | --- | --- | --- |
| 1 | 182 | 23.9 | 0.244 | 0.879 |
| 2 | 223 | 29.3 | 0.290 | 0.839 |
| 3 | 161 | 21.2 | 0.211 | 0.846 |
| 4 | 122 | 16.1 | 0.161 | 0.818 |
| 5 | 72 | 9.5 | 0.094 | 0.879 |

Modal n and percentages are based on N=760. Class labels were assigned descriptively after inspection of estimated trajectories. Modal class assignment was defined by the highest individual posterior probability.

## Supplementary Table S3. Availability of longitudinal immune-inflammatory measurements

| **Number of observed time points** | **Patients, n** |
| --- | --- |
| 2 | 24 |
| 3 | 188 |
| 4 | 548 |

All 760 patients contributed at least two observed time points to the observed-data LCMM likelihood.

## Supplementary Table S4. Pairwise Spearman correlations among NLR, SII, SIRI, and hs-CRP

| **Time** | **Marker 1** | **Marker 2** | **N** | **Spearman rho** | **P value** |
| --- | --- | --- | --- | --- | --- |
| Admission | NLR | SII | 759 | 0.935 | <0.001 |
| Admission | NLR | SIRI | 759 | 0.946 | <0.001 |
| Admission | NLR | hs-CRP | 759 | 0.717 | <0.001 |
| Admission | SII | SIRI | 759 | 0.883 | <0.001 |
| Admission | SII | hs-CRP | 759 | 0.682 | <0.001 |
| Admission | SIRI | hs-CRP | 759 | 0.721 | <0.001 |
| 24 h | NLR | SII | 702 | 0.965 | <0.001 |
| 24 h | NLR | SIRI | 702 | 0.969 | <0.001 |
| 24 h | NLR | hs-CRP | 702 | 0.836 | <0.001 |
| 24 h | SII | SIRI | 702 | 0.934 | <0.001 |
| 24 h | SII | hs-CRP | 702 | 0.819 | <0.001 |
| 24 h | SIRI | hs-CRP | 702 | 0.837 | <0.001 |
| 72 h | NLR | SII | 721 | 0.977 | <0.001 |
| 72 h | NLR | SIRI | 721 | 0.979 | <0.001 |
| 72 h | NLR | hs-CRP | 721 | 0.859 | <0.001 |
| 72 h | SII | SIRI | 721 | 0.956 | <0.001 |
| 72 h | SII | hs-CRP | 721 | 0.852 | <0.001 |
| 72 h | SIRI | hs-CRP | 721 | 0.864 | <0.001 |
| Day 7 | NLR | SII | 622 | 0.971 | <0.001 |
| Day 7 | NLR | SIRI | 622 | 0.973 | <0.001 |
| Day 7 | NLR | hs-CRP | 622 | 0.842 | <0.001 |
| Day 7 | SII | SIRI | 622 | 0.947 | <0.001 |
| Day 7 | SII | hs-CRP | 622 | 0.833 | <0.001 |
| Day 7 | SIRI | hs-CRP | 622 | 0.847 | <0.001 |

Correlations were calculated separately at admission, 24 h, 72 h, and day 7 using available paired observations.

## Supplementary Table S5. Bias-reduced sensitivity analyses for 90-day mRS 3–6

| **Analysis** | **N** | **Events** | **LCMM class** | **Class label** | **Bias-reduced OR (95% CI)** | **P value** |
| --- | --- | --- | --- | --- | --- | --- |
| Overall | 751 | 333 | 2 | Transient moderate response | 0.94 (0.57–1.55) | 0.817 |
| Overall | 751 | 333 | 3 | Delayed recovery | 1.35 (0.79–2.31) | 0.280 |
| Overall | 751 | 333 | 4 | Persistent high inflammation | 2.42 (1.29–4.52) | 0.006 |
| Overall | 751 | 333 | 5 | Extreme persistent high inflammation | 13.61 (2.34–79.26) | 0.004 |
| Exclude infection within 7 d | 651 | 259 | 2 | Transient moderate response | 0.93 (0.55–1.59) | 0.793 |
| Exclude infection within 7 d | 651 | 259 | 3 | Delayed recovery | 1.35 (0.76–2.41) | 0.302 |
| Exclude infection within 7 d | 651 | 259 | 4 | Persistent high inflammation | 2.48 (1.26–4.89) | 0.009 |
| Exclude infection within 7 d | 651 | 259 | 5 | Extreme persistent high inflammation | 26.28 (1.45–477.68) | 0.027 |
| Exclude sICH or malignant edema | 653 | 249 | 2 | Transient moderate response | 0.85 (0.50–1.44) | 0.548 |
| Exclude sICH or malignant edema | 653 | 249 | 3 | Delayed recovery | 1.24 (0.70–2.18) | 0.460 |
| Exclude sICH or malignant edema | 653 | 249 | 4 | Persistent high inflammation | 2.24 (1.15–4.37) | 0.017 |
| Exclude sICH or malignant edema | 653 | 249 | 5 | Extreme persistent high inflammation | 27.01 (1.46–500.47) | 0.027 |
| No reperfusion therapy | 338 | 129 | 2 | Transient moderate response | 0.47 (0.24–0.93) | 0.031 |
| No reperfusion therapy | 338 | 129 | 3 | Delayed recovery | 0.93 (0.46–1.91) | 0.850 |
| No reperfusion therapy | 338 | 129 | 4 | Persistent high inflammation | 1.33 (0.56–3.14) | 0.517 |
| No reperfusion therapy | 338 | 129 | 5 | Extreme persistent high inflammation | 1.89 (0.06–57.06) | 0.714 |
| Reperfusion therapy | 413 | 204 | 2 | Transient moderate response | 2.86 (1.19–6.88) | 0.019 |
| Reperfusion therapy | 413 | 204 | 3 | Delayed recovery | 2.90 (1.14–7.39) | 0.026 |
| Reperfusion therapy | 413 | 204 | 4 | Persistent high inflammation | 5.92 (2.11–16.65) | <0.001 |
| Reperfusion therapy | 413 | 204 | 5 | Extreme persistent high inflammation | 31.33 (4.66–210.50) | <0.001 |
| Complete 90-day follow-up | 751 | 333 | 2 | Transient moderate response | 0.94 (0.57–1.55) | 0.817 |
| Complete 90-day follow-up | 751 | 333 | 3 | Delayed recovery | 1.35 (0.79–2.31) | 0.280 |
| Complete 90-day follow-up | 751 | 333 | 4 | Persistent high inflammation | 2.42 (1.29–4.52) | 0.006 |
| Complete 90-day follow-up | 751 | 333 | 5 | Extreme persistent high inflammation | 13.61 (2.34–79.26) | 0.004 |

Low-stable recovery was the reference class. Bias-reduced logistic regression was used because the extreme persistent-high class showed near-separation for the binary outcome. Restricted analyses were considered sensitivity analyses rather than independent confirmatory models.

## Supplementary Table S6. Agreement between the LCMM-5 solution and 0–72 h K-means sensitivity classification

| **0–72 h K-means class** | **LCMM class 1** | **LCMM class 2** | **LCMM class 3** | **LCMM class 4** | **LCMM class 5** |
| --- | --- | --- | --- | --- | --- |
| 1 | 152 | 15 | 0 | 0 | 0 |
| 2 | 9 | 171 | 54 | 0 | 0 |
| 3 | 0 | 6 | 85 | 63 | 0 |
| 4 | 0 | 0 | 1 | 45 | 63 |

The k-means analysis was a secondary unsupervised robustness analysis restricted to patients with complete 0–72 h inflammatory measurements.

## Supplementary Table S7. Global interaction test for reperfusion therapy

| **Subgroup category** | **P for interaction** | **LR chi-square** | **df** | **Note** |
| --- | --- | --- | --- | --- |
| Reperfusion therapy | 0.017 | 12.03 | 4 | Exploratory. |

The exposure-by-reperfusion interaction was exploratory because treatment was nonrandom and the extreme persistent-high class was sparse in some strata.

## Supplementary Table S8. Paired-bootstrap changes in prediction performance

| **Comparison** | **Delta AUC** | **Delta AUC 95% CI low** | **Delta AUC 95% CI high** | **Delta Brier** | **Delta Brier 95% CI low** | **Delta Brier 95% CI high** | **Bootstrap resamples** |
| --- | --- | --- | --- | --- | --- | --- | --- |
| Clinical + admission NLR/SII minus Clinical model | 0.011 | 0.000 | 0.023 | -0.006 | -0.012 | -0.001 | 5000 |
| Clinical + 0-24 h immune change minus Clinical model | 0.000 | -0.004 | 0.005 | -0.000 | -0.002 | 0.001 | 5000 |
| Clinical + LCMM-5 minus Clinical model | 0.013 | 0.003 | 0.023 | -0.007 | -0.012 | -0.002 | 5000 |
| Clinical + LCMM-5 minus Clinical + admission NLR/SII | 0.002 | -0.010 | 0.012 | -0.001 | -0.006 | 0.005 | 5000 |

Positive delta AUC indicates higher discrimination; negative delta Brier indicates lower prediction error. All comparisons used the same complete-case prediction population.

## Supplementary Table S9. Operational LCMM class-assignment rule and estimated trajectory centroids

No fixed biomarker thresholds or deterministic if/then rules were used for class assignment. After the five-class LCMM was fitted, each patient was assigned to the class with the highest subject-specific posterior probability calculated from the observed longitudinal composite-burden measurements. The time-specific values below are fitted class mean composite-burden z scores and are descriptive centroids, not classification cutoffs. To reproduce preprocessing, the pooled log1p mean/SD pairs used for z-standardization were 2.032938/0.598496 for NLR, 7.301266/0.735354 for SII, 1.577285/0.709501 for SIRI, and 2.650096/0.934029 for hs-CRP. In the selected five-class model, the common random-intercept SD was 0.230999 and the residual SD was 0.275055; estimated class probabilities are reported in Supplementary Table S2. Posterior probabilities were calculated from the observed-data likelihood under the model specified in the Methods.

| **Class and descriptive pattern** | **Modal n (%)** | **Average posterior probability** | **Admission** | **24 h** | **72 h** | **Day 7** |
| --- | --- | --- | --- | --- | --- | --- |
| Low-stable recovery Low throughout | 182 (23.9%) | 0.879 | -0.968 | -0.847 | -0.929 | -1.070 |
| Transient moderate response Early rise, then decline | 223 (29.3%) | 0.839 | -0.613 | -0.005 | -0.379 | -0.776 |
| Delayed recovery Rise through 72 h, partial recovery by day 7 | 161 (21.2%) | 0.846 | -0.300 | 0.397 | 0.654 | 0.138 |
| Persistent high inflammation Persistently high | 122 (16.1%) | 0.818 | 0.313 | 1.088 | 1.408 | 1.008 |
| Extreme persistent high inflammation Persistently very high | 72 (9.5%) | 0.879 | 0.939 | 1.849 | 2.082 | 1.924 |

## Supplementary Table S10. Serial immune-inflammatory markers across LCMM-5 classes

Values are median (IQR). These marker-level distributions were moved from the main Table 2 to improve clinical readability while retaining the full longitudinal source summaries.

| **Marker / time** | **Overall** | **Class 1 Low-stable** | **Class 2 Transient moderate** | **Class 3 Delayed recovery** | **Class 4 Persistent high** | **Class 5 Extreme persistent high** | **P value** |
| --- | --- | --- | --- | --- | --- | --- | --- |
| Admission NLR | 5.00 (3.75, 7.75) | 3.35 (2.84, 4.17) | 4.52 (3.69, 5.43) | 5.53 (4.63, 7.34) | 8.73 (7.25, 11.14) | 14.40 (12.24, 17.40) | <0.001 |
| 24 h NLR | 7.43 (4.74, 12.04) | 3.27 (2.86, 3.98) | 6.62 (5.67, 7.93) | 8.82 (7.24, 10.32) | 14.46 (11.85, 16.36) | 24.02 (19.84, 29.27) | <0.001 |
| 72 h NLR | 6.26 (3.74, 12.72) | 2.90 (2.48, 3.57) | 4.99 (4.05, 5.89) | 10.06 (7.95, 11.89) | 15.67 (12.35, 19.70) | 25.81 (21.38, 31.67) | <0.001 |
| Day 7 NLR | 4.92 (3.17, 9.50) | 2.75 (2.34, 3.35) | 3.55 (3.08, 4.41) | 6.80 (5.58, 8.09) | 12.12 (10.23, 14.87) | 21.04 (17.23, 27.18) | <0.001 |
| Admission SII | 1141.0 (799.3, 1769.7) | 763.5 (608.9, 941.6) | 984.2 (772.5, 1253.8) | 1256.0 (1000.3, 1599.6) | 2046.8 (1582.6, 2613.9) | 3136.2 (2531.2, 3920.1) | <0.001 |
| 24 h SII | 1694.3 (1033.8, 2717.2) | 736.6 (610.2, 895.6) | 1497.2 (1249.9, 1914.9) | 1978.3 (1575.0, 2386.8) | 3323.7 (2680.9, 3995.8) | 5412.7 (4546.8, 6928.1) | <0.001 |
| 72 h SII | 1525.8 (850.4, 3157.1) | 659.3 (537.6, 800.4) | 1121.1 (905.3, 1397.4) | 2312.9 (1859.9, 2956.9) | 3887.5 (3012.2, 5030.3) | 6401.2 (5604.6, 7744.8) | <0.001 |
| Day 7 SII | 1187.8 (754.8, 2415.1) | 644.3 (519.3, 812.9) | 862.9 (699.4, 1025.2) | 1701.9 (1413.5, 2027.9) | 3283.4 (2607.2, 3823.3) | 5720.0 (4623.7, 7252.4) | <0.001 |
| Admission SIRI | 2.48 (1.73, 4.29) | 1.48 (1.18, 2.00) | 2.25 (1.73, 2.63) | 2.86 (2.10, 3.87) | 5.20 (3.98, 6.60) | 9.21 (7.13, 10.81) | <0.001 |
| 24 h SIRI | 4.17 (2.50, 7.82) | 1.64 (1.29, 2.08) | 3.55 (2.88, 4.63) | 5.22 (3.99, 6.58) | 10.32 (7.75, 12.84) | 20.03 (16.04, 26.03) | <0.001 |
| 72 h SIRI | 3.60 (1.85, 9.36) | 1.29 (1.02, 1.74) | 2.47 (1.97, 3.17) | 6.25 (4.92, 8.03) | 12.44 (9.75, 15.51) | 24.62 (17.76, 30.68) | <0.001 |
| Day 7 SIRI | 2.50 (1.38, 6.05) | 1.19 (0.96, 1.47) | 1.65 (1.26, 2.20) | 3.80 (2.88, 4.58) | 8.43 (6.83, 11.14) | 16.86 (13.59, 21.37) | <0.001 |
| Admission hs-CRP, mg/L | 6.87 (4.14, 11.27) | 3.35 (2.50, 4.61) | 5.92 (4.28, 8.36) | 7.42 (5.59, 10.25) | 13.70 (10.38, 18.50) | 22.78 (18.95, 29.30) | <0.001 |
| 24 h hs-CRP, mg/L | 15.82 (8.24, 29.61) | 5.09 (3.86, 7.25) | 12.58 (9.42, 17.02) | 19.30 (14.67, 25.41) | 36.22 (27.19, 47.65) | 59.37 (48.29, 79.51) | <0.001 |
| 72 h hs-CRP, mg/L | 16.45 (8.40, 43.84) | 6.16 (4.85, 8.78) | 10.47 (7.78, 14.16) | 28.67 (21.05, 38.28) | 58.11 (43.92, 75.44) | 92.46 (78.41, 120.98) | <0.001 |
| Day 7 hs-CRP, mg/L | 10.34 (5.20, 27.65) | 4.49 (3.40, 6.12) | 6.43 (4.54, 8.64) | 17.68 (12.52, 23.46) | 47.34 (27.79, 62.29) | 90.19 (64.30, 151.48) | <0.001 |

# Supplementary Figures

## Supplementary Figure S1. LCMM model selection and selected five-class trajectory profiles.

Panel A compares model-selection criteria across candidate class numbers; panel B shows the estimated mean composite-burden trajectories for the selected five-class solution.

## Supplementary Figure S2. Correlation matrices for serial inflammatory markers.

Spearman correlation matrices are shown separately for admission, 24 h, 72 h, and day 7 measurements.

## Supplementary Figure S3. Crosswalk between LCMM-5 and 0–72 h K-means sensitivity classifications.

Cell values are patient counts among those with complete 0–72 h inflammatory data used in the k-means sensitivity analysis.

## Supplementary Figure S4. Restricted-sample bias-reduced sensitivity analyses.

Forest plots show bias-reduced adjusted odds ratios for persistent-high and extreme persistent-high classes across restricted analyses. Exact estimates are reported in Supplementary Table S5.
